# Supplementary material for: Receipt of healthcare provider advice to quit tobacco use among Indian men
Source: PLoS One. 2026 May 18;21(5):e0349022. doi: 10.1371/journal.pone.0349022 (PMC13183209; doi:10.1371/journal.pone.0349022)
Supplement: S1 File — (DOCX) [file pone.0349022.s001.docx]

**Supplementary file 1**

**S1 Table:** Variable transformation sheet

| **Dependent Variables** | | | |
| --- | --- | --- | --- |
| **Original source variables from NFHS surveys** |  | **Study variables created from source variables** | |
| **Source Variables** | **Responses** | **Variables** | **Response categories** |
| In the last 12 months, have you visited a doctor or other healthcare provider | Yes  No | **Receipt of HCP advice to quit*:** Cigarette/Bidi user visited healthcare was advised to quit smoking. | - Received quit advice - Did not receive quit advice |
| During any of these visits, were you advised to quit smoking or using tobacco in any other form | Yes  No |  |  |
| **Explanatory Variables** | | | |
| Age of the individual in completed years |  | Age groups | - 15-29 years - 30-44 years - 45 to 54 years |
| Occupation grouped | Not working  Professional/technical/managerial  Clerical  Sales  Services/household and domestic  Agricultural  Skilled and unskilled manual  Other  Don’t know | Occupation group | - Unemployed - jobs in the sectors of agriculture, unskilled/skilled manual jobs, and domestic jobs - Jobs in professional, clerical, and sales sector. |
| Education level | No Education  Primary  Secondary  Higher | Education level | - No Education - Primary - Secondary - Higher |
| Wealth index Combined | Poorest  Poorer  Middle  Richer  Richest | Household Wealth Quintile | - Poorest - Poorer - Middle - Richer - Richest |
| Do you belong to a scheduled caste, a scheduled tribe, other backward castes, or none of these | Scheduled Caste  Scheduled tribe  OBC  None of them | Caste | - SC - ST - OBC - Others |
| On average, how many cigarettes do you currently smoke each day? |  | Frequency of smoking | - ≤ 10 cigarettes/bidi per day - > 10 cigarettes/bidi per day |
| On average, how many bidis do you currently smoke each day? |  |  |  |
| Uses Khaini | Yes  No | Current use of smokeless tobacco | - Yes - No |
| Uses Paan with tobacco | Yes  No |  |  |
| Do you currently have diabetes | Yes  No | Previously diagnosed with an NCD | - Yes - No |
| Do you currently have Chronic respiratory diseases including Asthma | Yes  No |  |  |
| Do you currently have any heart disease? | Yes  No |  |  |
| Do you currently have cancer? | Yes  No |  |  |
| Ever undergone an oral cavity examination/ Ever undergone an oral cavity examination for oral cancer | Yes  No | Oral examination | - Yes - No |
| In the last 30 days, did someone smoke in your home or anywhere else when you were present | Yes  No | Second hand smoke | - Yes - No |
| Do you drink alcohol | Yes  No | Alcohol consumption status | - Yes - No |
