# Supplementary material for: Receipt of healthcare provider advice to quit tobacco use among Indian men
Source: PLoS One. 2026 May 18;21(5):e0349022. doi: 10.1371/journal.pone.0349022 (PMC13183209; doi:10.1371/journal.pone.0349022)
Supplement: S2 File — (DOCX) [file pone.0349022.s002.docx]

**Supplementary File 2:** State level differences in the receipt of HCP advice to quit among combustible tobacco users

| **State** | **NFHS 4** | **NFHS 5** |
| --- | --- | --- |
|  | **Prevalence (95% CI)** | **Prevalence (95% CI)** |
| Andaman & Nicobar Islands | 70.0 (44.4-87.2) | 75.6 (24.7-96.7) |
| Andhra Pradesh | 72.3 (60.7-81.5) | 61.9 (48.7-73.6) |
| Arunachal Pradesh | 29.4 (18.7-43.0) | 57.6 (47.9-66.7) |
| Assam | 34.6 (28.0-41.8) | 37.4 (29.9-45.5) |
| Bihar | 60.8 (49.9-70.7) | 56.6 (42.2-70.0) |
| Chandigarh^#^ | - | - |
| Chhattisgarh | 59.1 (48.7-68.8) | 56.5 (43.5-68.6) |
| Dadra & Nagar Haveli | 47.4 (11.1-86.6) | 85.6 (56.0-96.5) |
| Daman & Diu* | 89.2 (55.3-98.2) | - |
| Goa^#^ | - | 83.8 (33.5-98.2) |
| Gujarat | 66.0 (56.1-74.6) | 48.8 (32.5-65.3) |
| Haryana | 62.0 (50.8-72.0) | 52.4 (41.9-62.7) |
| Himachal Pradesh | 61.5 (51.9-70.2) | 61.4 (38.9-79.8) |
| Jammu & Kashmir | 54.9 (49.9-59.7) | 42.3 (35.2-49.8) |
| Jharkhand | 50.7 (38.6-62.8) | 34.7 (23.6-47.8) |
| Karnataka | 75.8 (66.0-83.5) | 48.1 (37.4-59.0) |
| Kerala | 53.1 (41.4-64.4) | 56.0 (42.9-68..4) |
| Madhya Pradesh | 52.5 (47.6-57.4) | 59.1 (50.7-67.1) |
| Maharashtra | 72.2 (57.6-83.3) | 63.4 (41.6-80.8) |
| Manipur | 39.2 (28.2-51.4) | 32.5 (18.4-50.6) |
| Meghalaya | 43.4 (33.9-53.4) | 56.6 (45.1-67.4) |
| Mizoram | 32.4 (23.4-43.0) | 41.6 (29.7-45.5) |
| Nagaland | 40.7 (28.5-54.1) | 24.1 (8.4-52.4) |
| Delhi | 52.9 (26.9-77.3) | 67.6 (54.4-78.5) |
| Odisha | 34.4 (28.0-41.4) | 37.8 (30.4-45.6) |
| Puducherry | 21.7 (4.5-61.9) | 61.5 (28.5-86.5) |
| Punjab | 75.2 (53.8-88.8) | 81.5 (68.0-90.1) |
| Rajasthan | 39.8 (33.2-46.8) | 46.2 (40.1-52.4) |
| Sikkim | 15.6 (5.7-36.4) | 43.5 (16.5-74.9) |
| Tamil Nadu | 54.3 (45.1-63.3) | 69.0 (56.1-79.5) |
| Tripura | 41.8 (34.1-49.9) | 72.0 (61.5-80.6) |
| Uttar Pradesh | 53.1 (48.6-57.5) | 50.6 (45.3-55.8) |
| Uttarakhand | 54.4 (43.9-64.6) | 32.4 (20.5-47.2) |
| West Bengal | 46.4 (39.6-53.4) | 61.1 (54.7-67.2) |
| Telangana | 53.8 (40.2-66.8) | 77.7 (69.5-84.1) |
| Ladakh* | - | 14.6 (5.5-33.2) |

HCP= Healthcare Provider; NFHS= National Family Health Survey; CI= Confidence Interval; ^#^Fewer samples in Chandigarh [NFHS-4 & NFHS-5] and Goa [NFHS-4]; *Daman & Diu was included in Dadra & Nagar Haveli in NFHS-5; *Ladakh was constituted as a region separate from Jammu & Kashmir in 2019-20.
