# Supplementary material for: Receipt of healthcare provider advice to quit tobacco use among Indian men
Source: PLoS One. 2026 May 18;21(5):e0349022. doi: 10.1371/journal.pone.0349022 (PMC13183209; doi:10.1371/journal.pone.0349022)
Supplement: Attachment — Submitted filename: Response to Reviewers.docx [file pone.0349022.s003.docx]

**Title:** Receipt of healthcare provider advice to quit tobacco use among Indian men

**Journal:** PLOS ONE

**Manuscript ID:** PONE-D-25-29325

**Point by point response to reviewer comments**

Reviewer #1: This manuscript addresses an important and understudied aspect of tobacco control in India—the receipt of healthcare provider (HCP) advice to quit among male smokers, using two large NFHS datasets (4 & 5). The topic aligns well with national and global tobacco control priorities, leveraging high-quality, nationally representative data.
The paper is clearly written, the results are relevant, and the public-health implications are important. However, there are methodological limitations, conceptual gaps, and missing clarifications that require attention.

**Author response:** We appreciate the reviewer’s comment. We revised the manuscript accordingly. Where suggested revisions were not feasible due to data limitations, we explicitly acknowledged these constraints and reported them in the limitations section.

Introduction
1. The NFHS questions regarding the HCP visit and advice
The definition does not specify which type of HCP (doctor, nurse, AYUSH, dentist, etc.). The study assumes HCPs are comparable. In the Indian context, it is a major oversimplification. Also the study could be biased as the information on the HCPs are based on the information from the patient.
It is ideal to categorise the HCPs and might give an input on the variations in the smokers in each category

**Author response:** We agree that disaggregating Healthcare provider (HCP) by type (e.g., doctors, nurses, AYUSH practitioners, dentists) and reporting advice to quit tobacco by provider category would add important nuance, particularly in the Indian context, where care-seeking is heterogeneous.

However, we are unable to undertake such an analysis due to inherent limitations of the National Family Health Survey (NFHS) data. Specifically, advice to quit tobacco use is captured only through the following linked items: (i) “In the last 12 months, have you visited a doctor or other healthcare provider?” and (ii) “During any of these visits, were you advised to quit smoking or using tobacco in any other form.” These items do not specify the type of HCP associated with the advice to quit.

While NFHS includes separate questions on interactions with specific provider types (such as doctors, nurses, or AYUSH providers), these items pertain to different recall periods (e.g., last 3 months) or distinct service domains (e.g., family planning or immunization). As a result, these variables could not be harmonized with the tobacco cessation advice variable in a methodologically valid manner. We have recognized this as a major limitation of our study in **line 350-352**.

2. Under the health status subheading, two questions are mentioned
“ever had oral cavity examination”, “ever undergone oral cancer screening”
These are not equivalent constructs, yet are pooled as one variable for oral cancer screening in the table. This could be seen as a methodological flaw. It can be mentioned as a limitation, or by analysing after excluding this variable

**Author response:** We agree with the reviewer that “ever had an oral cavity examination” and “ever undergone oral cancer screening” are not strictly equivalent constructs. As the reviewer correctly notes, NFHS-4 captured oral examination using a broader item, whereas NFHS-5 introduced a more specific question on oral cancer screening.

To maintain comparability across survey rounds and avoid excluding a potentially relevant oral health–related variable, we retained this measure and operationalized it conservatively as “ever had an oral examination.” We acknowledge that this variable likely captures a heterogeneous set of encounters, ranging from general oral examinations to formal oral cancer screening, and therefore should not be interpreted as a precise measure of oral cancer screening uptake.

We considered excluding this variable; however, doing so would have omitted information on prior oral health contact, which may plausibly influence healthcare provider–initiated tobacco cessation advice, particularly in a high-tobacco-burden setting such as India. To minimize misinterpretation, we have avoided referring to this variable as “oral cancer screening” and have explicitly acknowledged the non-equivalence of constructs and potential misclassification as a study limitation **(Lines 354–357)**.

Methods
3. NFHS 4 mentions 4 NCDs, and NFHS 5 mentions 6 NCDs; pooling these NCDs can falsely increase the association. Were any corrections made on the same?

**Author response:** To ensure comparability, we restricted the NCD variable to the four conditions common to both survey rounds (diabetes, asthma, heart disease, and cancer). We revised our analysis by applying the updated variable for ‘Diagnosed with an NCD’ and made corresponding changes to our results section **[line 276-282]** and **table 2** in the revision.

Results:
4. Figure 1 shows many determinants for the advice to quit. However potential confounders are missed in analysis such as: Frequency of smoking or cigarettes/bidis per day (Heavier smokers are more likely to mention it and to be advised to quit).; Number of healthcare visits (More visits → higher probability of receiving advice); Type of facility (public vs private); Quit advice frequency differs widely across sectors in India. Provider characteristics (gender, training, cadre), although there should not be a difference, some people tend to listen to a male HCP more.

**Author response:** We developed our conceptual framework (figure 1), by reviewing published studies, NFHS survey questionnaires and respective data sets. Based on your suggestion, we included the item ‘Frequency of smoking cigarettes/bidi per day’ by developing it from independent items capturing ‘number of cigarettes smoked per day’ and ‘number of bidis smoked per day’. We captured it as a binary item with responses ‘≤ 10 cigarettes/bidi per day’ and ‘>10 cigarettes/bidi’ per day ([Cigarette Smoking Reduction and Health Risks: A Systematic Review and Meta-analysis - PubMed](https://pubmed.ncbi.nlm.nih.gov/32803250/)). We revised our Figure 1, Table 1, Table 2, Results and discussion by including this important variable.

With respect to the items such as number of healthcare visits, type of facility, and provider characteristics, we are unable to include them since the NFHS surveys did not capture these items. We have acknowledged this in the limitations, and indicated the need for future studies by including these items in **lines 357-359**.

5. Needs clarity on tables and results, need a bit organizing

**Author response:** We revised and better organized the tables and Results section in line with the suggested corrections to improve clarity. Specifically, in Table 2, we now present unweighted frequencies for all independent variables alongside weighted percentages for the outcome variable (i.e., receipt of healthcare provider advice to quit), improving clarity and interpretability.

Minor Issues

1. Abstract is strong but could briefly mention limitations.

**Author response:** Thank you we have reported the study limitations in the abstract of the manuscript **(line 53 & 54)**.

2. Improve grammar & discussion to reduce repetition.

**Author response:** We improved grammar and reduced repetition in the discussion.
