# Supplementary material for: Receipt of healthcare provider advice to quit tobacco use among Indian men
Source: PLoS One. 2026 May 18;21(5):e0349022. doi: 10.1371/journal.pone.0349022 (PMC13183209; doi:10.1371/journal.pone.0349022)
Supplement: Attachment — Submitted filename: Response to Review comments 3.20.26.docx [file pone.0349022.s004.docx]

**Point by point response to review comments**

**Title:** Receipt of healthcare provider advice to quit tobacco use among Indian men

**Journal:** PLOS ONE

**Manuscript ID:** PONE-D-25-29325

Reviewer #2: The current study explores the important topic of tobacco-cessation counseling by way
of HCP advice. The use of large-scale representative data, the statistical analysis and
the results are acceptable. However, there are **gaps in discussion** that need to be addressed to
make clear recommendations. The points below are noted for the consideration of the
authors.

**Author response:** Dear Reviewer, thank you for your careful review of our manuscript and the suggestions you have made. We agree with the revisions suggested and had made the corrections in the manuscript. We ensured to include the suggested articles in our discussion and strengthen our findings and their interpretation by including a state-level differences in receipt of HCP advice to quit as a supplementary file. We look forward to your positive consideration of our manuscript.

1. Ref: Rayhan Rahman, Illias Sheikh, Yash Merchant, Ankita Mathur, Toufiq Noor, Vini Mehta,
Oral cancer screening in India: a secondary analysis of disparities using national family health survey data, Oral Oncology, Volume 169, 2025, 107632, ISSN 1368-8375, https://doi.org/10.1016/j.oraloncology.2025.107632.
(https://www.sciencedirect.com/science/article/pii/S1368837525004610)

The above study uses NFHS data and explores an important point overlooked in the current study- the prevalence of tobacco usage- smoke/smokeless differs widely between states as observed in the NFHS survey too.

It mentions: “Screening participation is directly related to lower tobacco prevalence in the population. The study found that people from high-tobacco-prevalence states were 42 % less likely to be screened (Adjusted Odds Ratio (AOR): 0.58, p < 0.01) than those from low-prevalence states”.

This finding has also been noted in the GATS study.

This wide variation between the Southern Indian states and Union territories as compared to Northern Indian states can be mentioned and even included in the analysis.

**Author response:**

Thank you for the suggestion. We have included the state-wise variations in HCP advise to quit as a supplementary file. We have discussed the variations in HCP advice to quit in connection with the observations made by Rahman et al (2025), and GATS India survey 2016-17. The changes were made to **lines 306 to 312** and **supplementary file 2** was added.

2.Ref:

https://www.researchgate.net/publication/368273529_Utilization_of_dental_care_services_among_adult_Indian_population_A_meta-analysis_of_evidence_from_2011-2022

The above is a meta-analysis that estimates dental care seeking among Indian adults and discusses various factors including the North – South divide in oral care seeking, findings worth noting and mentioning in the discussion.
**Author response:** Thank you for the suggestion. We have revised our discussion section to include the suggested study in the discussion section in lines **350-353.**

3. The National Oral Health program launched by the MOHFW in 2014 and its scope could be included in the discussion.

**Author response:** Thank you for the suggestion. We have incorporated a discussion on the scope of the National Oral Health Programme in the revised manuscript (**Lines 348–353**).

4. An important finding from NFHS 4 vs NFHS 5- as noted in a recent study (referenced below) is that “The proportion of men reporting oral cavity examinations was 9% (out of 112,122 interviewed men) in NFHS-4, which reduced to 0.2% (out of 716,443 interviewed men) in NFHS-5”

Ref: Learning from the Indian National Family Health Survey to assess population based oral, cervix and breast cancer screening in low-and-middle income countries. Borkotoky, Kakoli et al.
The Lancet Regional Health - Southeast Asia, Volume 30, 100483

**Author response:** Thank you for the suggestion, we have included the suggested study in our discussion in **lines 345-346**

5. Ref: Ramanadhan S, Xuan Z, Choi J, Mahtani SL, Minsky S, Gupte H, Mandal G, Jagiasi D, Viswanath K. Associations between sociodemographic factors and receiving "ask and advise" services from healthcare providers in India: analysis of the national GATS-2 dataset. BMC Public Health. 2022 Nov 18;22(1):2115. doi: 10.1186/s12889-022-14538-2. PMID: 36401241; PMCID: PMC9673333.

The findings of the above study, analyzing the GATS-2 dataset, are relevant and would do well to be included in the discussion for the current study

**Author response:** Thank you for the suggestion, we have included the suggested study in our discussion.

6. The fact that the timing of the NFHS 5 survey was during the pandemic when rampant COVID screening and testing were underway could have impacted health seeking and also contributed to the noticed increase in self-reports of oral examinations during NFHS 5 may be worth mentioning.

**Author response:** Thank you for this important observation. We agree that the timing of NFHS-5 during the COVID-19 pandemic may have influenced health-seeking behaviour, oral examinations, and interactions with healthcare providers. We have acknowledged this as a limitation of the study in the revised manuscript (**Lines 378–379**)**.**

Reviewer #3: Its a nice piece of work, done on a very pertinent and buring topic of the contemprory times. However, certain aspects needs clarificatrion.

1. The study describes that only 50% of tobaco users attending the health care facility were given advice to quit. In this context it is pertinent to note the primery reason for attending the health care facility. Since it will have a large bearing on giving advice. For instance in case of accidents or emergencies it is unlikely that HCP will provide advice to quit. They will be focused more on stabilizing the patients. Same is the case with attendence for resons which are unrelated to tobacco use, emaple - Surgical conditions etc.

**Author response:** Thank you for this valuable observation. We agree that the primary reason for visiting a healthcare facility can significantly influence the likelihood of receiving advice to quit tobacco. However, the NFHS surveys do not capture information on the reason for healthcare visits. We have acknowledged this as a limitation in the revised manuscript (**Lines 375–378**) and have interpreted our findings with appropriate caution**.**

2. Findings will be more reliable if data is provided to demonstarte that the visit to health facility was due to a conditon/ health state which arised due to tobacco use. since such information is not availabe in present study, policy impications becomes unjustifiable.

**Author response:** Thank you for this important comment. We acknowledge that the dataset does not include information on the specific reasons for healthcare visits, and therefore we are unable to ascertain whether these visits were related to tobacco use. This has been explicitly recognized as a limitation in the revised manuscript [**lines 375 to 378**].

7. PLOS authors have the option to publish the peer review history of their article ([what does this mean?](https://journals.plos.org/plosone/s/editorial-and-peer-review-process#loc-peer-review-history)). If published, this will include your full peer review and any attached files.

If you choose “no”, your identity will remain anonymous but your review may still be made public.

**Do you want your identity to be public for this peer review?** For information about this choice, including consent withdrawal, please see our [Privacy Policy](https://www.plos.org/privacy-policy).

Reviewer #2: No

Reviewer #3: No

[NOTE: If reviewer comments were submitted as an attachment file, they will be attached to this email and accessible via the submission site. Please log into your account, locate the manuscript record, and check for the action link "View Attachments". If this link does not appear, there are no attachment files.]

To ensure your figures meet our technical requirements, please review our figure guidelines: https://journals.plos.org/plosone/s/figures

You may also use PLOS’s free figure tool, NAAS, to help you prepare publication quality figures: https://journals.plos.org/plosone/s/figures#loc-tools-for-figure-preparation.

NAAS will assess whether your figures meet our technical requirements by comparing each figure against our figure specifications.
